# Indirect resin composites

**DOI:** 10.4103/0972-0707.73377

**Published:** 2010

**Authors:** Suresh Nandini

**Affiliations:** Department of Conservative Dentistry, Meenakshi Ammal Dental College, Chennai - 600 095, India

**Keywords:** Composite inlays, fiber-reinforced composites, indirect resin composites

## Abstract

Aesthetic dentistry continues to evolve through innovations in bonding agents, restorative materials, and conservative preparation techniques. The use of direct composite restoration in posterior teeth is limited to relatively small cavities due to polymerization stresses. Indirect composites offer an esthetic alternative to ceramics for posterior teeth. This review article focuses on the material aspect of the newer generation of composites. This review was based on a PubMed database search which we limited to peer-reviewed articles in English that were published between 1990 and 2010 in dental journals. The key words used were ‘indirect resin composites,’ composite inlays,’ and ‘fiber-reinforced composites.’

## INTRODUCTION

Dental composite formulations have been continuously evolving ever since Bis-GMA was introduced to dentistry by Bowen in 1962. Recent developments in material science technology have considerably improved the physical properties of resin-based composites and expanded their clinical applications. Dental restorative composite materials can be divided into direct and indirect resin composites (IRC). IRCs are also referred to as prosthetic composites or laboratory composites. These materials offer an esthetic alternative for large posterior restorations. There are a plethora of materials available nowadays.

## THE NEED FOR IRC

Dental resin composites were introduced initially for use as anterior restorative materials. Later, with technological improvements, the prospect of restoring posterior teeth with composite was introduced. Though there are numerous causes for failure of clinical restorations made of direct composites, the major cause with the earlier posterior composites was poor wear resistance.[1,2] While the newest direct composite resins offer excellent optical and mechanical properties, their use in larger posterior restorations is still a challenge since polymerization shrinkage remains a concern in cavities with high C-factor. Though there have been numerous advances in adhesive systems, it is observed that the adhesive interface is unable to resist the polymerization stresses in enamel-free cavity margins.[3,4] This leads to improper sealing, which results in microleakage, postoperative sensitivity, and recurrent caries. The achievement of a proper interproximal contact and the complete cure of composite resins in the deepest regions of a cavity are other challenges related to direct composite restorations. Various approaches have been developed to improve some of the deficiencies of direct-placement composites.[5,6] However, no method has completely eliminated the problem of marginal microleakage associated with direct composite.[4,7] IRCs were introduced to reduce polymerization shrinkage and improve the properties of material.

Though the mechanical properties of the IRCs are much inferior to that of ceramics, in some clinical situations, IRCs can supplement and complement (rather than replace) ceramic restorations: for example, in coronal restoration of dental implants. As ceramics exhibit a high modulus of elasticity and absorb little of the masticatory energy, considerable amount of the masticatory force is transmitted to the implant and the periosseous structure, reducing the longevity of the restoration. Polymers become the materials of choice in this situation because they absorb relatively more of the occlusal stress. For patients with poor periodontal structures who require occlusal coverage, stress-absorbing materials like IRCs are indicated[8]

This review article focuses on the material aspect of this newer generation of composites. This review was based on a PubMed database search that we limited to peer-reviewed English-language articles published between 1990 and 2010 in dental journals. For the literature search the key words used were ‘indirect resin composites,’ ‘composite inlays,’ and ‘fiber-reinforced composites.’

## TYPES OF IRCS

Touati and Mörmann introduced the first generation of IRCs for posterior inlays and onlays in the 1980s.[9] Direct resin composites were composed mostly of organic resin matrix, inorganic filler, and coupling agent. The first-generation IRCs had a composition identical to that of the direct resin composite marketed by the same manufacturer and the materials also bore names similar to that of the direct materials.

Upon light initiation, camphoroquinone decomposes to form free radicals and initiates polymerization, resulting in the formation of a highly crosslinked polymer. It is observed that 25%–50% of the methacrylate group remains unpolymerized.[10]

For inlay composites, an additional or secondary cure is given extraorally, which improves the degree of conversion and also reduces the side effects of polymerization shrinkage. The only shrinkage that is unavoidable is that of the luting cement.[11] It was observed that the first-generation IRCs showed improved properties only in lab studies but had failures in clinical studies. With the first-generation composites either a direct–indirect /semi-indirect method or an indirect method was used to fabricate the restoration.[12]

### Direct-indirect/semi-indirect method

The composite material is condensed into the cavity after the separating medium is applied to the cavity. This separating medium helps in easy removal of the inlay after the initial intraoral curing. The restoration is then subjected to extraoral light or heat tempering in an oven. DI-500^®^ Oven (Coltene Whaledent) or a Cerinate^®^ Oven (Den-Mat Corp) can be used at 110°C for 7 min. This technique eliminates the need for an impression of the cavity and the procedure can be completed in a single sitting.[12] Brilliant DI^®^ (Coltene Whaledent) and True Vitality^®^ (Den-Mat Corp) are examples of material that uses both light and heat for this technique.

### Indirect

The inlay is fabricated in a die. After the separating medium is applied to the die, composite material is condensed in increments into the cavity and light cured for 40 sec for each surface. The inlay is then removed and heat cured in an oven at 100°C for 15 min (CRC-100 Curing Oven^®^, Kuraray). The advantage of this technique is that the proximal contours can be achieved appropriately. One of the first materials introduced by Ivoclar was SR-Isosit^®^, which was marketed as Concept^®^ in the US. This system uses a hydropneumatic heat cure in the Ivomat^®^ apparatus. The polymerization takes place in water at 120°C and a pressure of 6 bar for 10 min.[13] Another example of indirect material is Clearfil CR Inlay^®^ (Kuraray),which uses light and heat for the indirect technique. Conquest^®^ (Jeneric/Pentron), EOS^®^ (Vivadent), and Dentacolor^®^ (Kulzer) use only heat for additional curing, whereas Visio-Gem^®^ (ESPE-Premiere) uses heat and vaccum for additional curing.[12] It is possible to use any posterior composite for indirect techniques with additional curing.

## PROPERTIES OF FIRST-GENERATION COMPOSITES

Various studies have demonstrated the properties of the first-generation composites. It was observed that the degree of conversion increased by 6%–44%. Flexural strength ranges from 10–60 MPa and elasticity modulus ranges from 2000–5000 MPa.[13–16] The effect of additional cure may vary among the different studies because certain materials respond better to additional cure and because different methodologies may have been employed for determining these parameters. Post-cure temperature had a much higher influence on the degree of conversion than post-cure duration. Wendt[17] demonstrated that a 5-min post-light-heat treatment at 123°C (253°F) increased the hardness and wear resistance by as much as 60%–70%. But, clinically, heat treatment did not influence the wear resistance of the clinical restorations. Regardless of time, the wear rates for the heat-treated and non-heat-treated resin restorations were exactly the same: around 60 *µ* in 3 years. Clinical studies of other compositions given the same heat treatment generated similar results.[18,19] It was observed that supplementing conventional photocure with additional cure increased the monomer conversion but did not necessarily improve the physical properties.[13]

## DISADVANTAGES OF FIRST-GENERATION COMPOSITES

First-generation composites showed poor *In vitro* and clinical performance. Deficient bonding between organic matrix and inorganic fillers was the main problem leading to unsatisfactory wear resistance, high incidence of bulk fracture, marginal gap, microleakage, and adhesive failure in the first attempts to restore posterior teeth. Measures to solve these problems included increasing of inorganic filler content, reduction of filler size, and modification of the polymerization system.

## SECOND-GENERATION IRC

The clinical failures endured with first-generation composites and the limitations faced with ceramic restorations led to the development of improved second-generation composites. The improvements occurred mainly in three areas: structure and composition, polymerization technique, and fiber reinforcement.[9]

### Structure and composition

The second-generation composites have a ‘microhybrid’ filler with a diameter of 0.04–1 *µ*, which is in contrast to that of the first-generation composites that were microfilled. The filler content was also twice that of the organic matrix in the latter composites. By increasing the filler load, the mechanical properties and wear resistance is improved, and by reducing the organic resin matrix, the polymerization shrinkage is reduced.[9] The new composite resins like Artglass^®^ and belleGlass HP^®^ contain high amounts of filler content, which make them adequate for restoring posterior teeth. Others, such as Solidex^®^ (Shofu Inc.), have intermediate filler loading, which enables better esthetics and are preferred for anterior tooth.[20,21]

### Polymerization techniques

Even additional light curing extraorally did not efficiently improve the degree of conversion. Thus, specific conditions like heat, vacuum, pressure, and oxygen-free environment are utilized for polymerization of second-generation IRCs.[22] The various techniques used for additional cure are desribed below.

#### Heat polymerization

The temperature usually used for IRC ranges from 120–140°C. Ideally, the temperature applied in this treatment must be above the composite’s glass transition temperature (Tg).[23] This allows a significant increase in polymer chain mobility, favoring additional cross-linking and stress relief.[24] Nevertheless, it is noteworthy that overheating may cause degradation of the composite. The heat can be applied in autoclaves, cast furnaces, or special ovens.[25] Post-cure heating of resin composite materials decreases the levels of unreacted monomer after the initial light-curing stage. Basically, two mechanisms can be involved in this phenomenon. First, the residual monomer would be covalently bonded to the polymer network as a result of the heat treatment, leading to increase in the conversion itself. Second, unreacted monomers would be volatilized during the heating process.[16] The combination of heat and light increases the thermal energy sufficiently to allow better double-bond conversion. This concept was first used by Heraeus-Kulzer in the development of Charisma^®^. It was observed that the wear resistance increased by 35% on curing with both light and heat when compared to curing with light only.[26]

#### Nitrogen atmosphere

Air, because it contains oxygen, tends to inhibit polymerization and also plays an important role in the apparent translucency or opacity of the cured resin restoration. Oxygen entrapment in the restoration tends to break up or diffract natural light as it reflects from the surface of the restoration. Removing all of the encased air causes the restoration to become considerably more translucent. Entrapped oxygen increases the wear rate by weakening the wall around it. Nitrogen pressure eliminates internal oxygen before the material begins to cure. This influences the degree of conversion, esthetics, wear, and abrasion.[8] BelleGlass^®^ and Sculpture Plus^®^ employ this method of curing in a nitrogen bell.

#### Soft start or sow curing

The concept of slow curing described by Mehl[26] is based upon the concept that a slower rate of curing will allow a greater level of polymerization. Faster rates of polymerization tend to prematurely rigidify the newly formed polymerized branches. Such a condition will increase their stiffness, disallowing further propagation of the molecule. Such a concept is incorporated in the curing process for both belleGlass^®^ and Cristobal^®^.

#### Electron beam irradiation

Electron beam irradiation is another method described for improving the composite’s properties.[27] This methodology is used with polymers like polyethylene, polycarbonate, or polysulfone.[28] The two main reactions that occur when a polymer is subjected to electron beam irradiation are chain breakage and chain linkage. When breakage of chains occurs at the region of entanglement, there is induction of dense packing. This influences the bond between the filler and matrix, thus improving the mechanical properties and increasing success rates. The possible disadvantage of this method is polymer degradation and discoloration of the resin. The radiation dosage usually given is 200 KGy, but lower dosage like 1 KGy also has been shown to improve the properties.[29] Due to economic reasons it is impossible to irradiate single crowns or FPDs. Behr and Rosentritt demonstrated that irradiated raw materials of composites can be mixed with new material to improve properties.

#### Fiber reinforcement

Fiber-reinforced composites were introduced by Smith in the 1960s. Polyethylene fibers,[30] carbon/graphite fibers, Kevlar^®^, and glass fibers[31–33] were tested. Glass and polyethylene are the commonly used fibers in dentistry. Fibers act as crack stoppers and enhance the proprety of composite. The resin matrix acts to protect the fiber and fix their geometrical orientation.[34,35] Boron oxide, a glass-forming agent is present at 6–9 wt% in E-fibers and <1 wt% in S-fibers. E- and S-fibers are the ones most commonly used in dentistry.[36,37] The details of the FRC are shown in Table 1a and 1b.[38,39] The fibers can be arranged in one direction (unidirectional), with the fibers running from one end to other in a parallel fashion. Alternatively, the fibers can be arranged in different directions to one another, resulting either in a weave- or mesh-type architecture.[34] When the directional orientation of the fiber long axis is perpendicular to the applied forces, it will result in strength reinforcement.[40] Forces that are parallel to the fiber orientation will produce matrix-dominated failures and consequently yield little reinforcement. Multidirectional reinforcement is accompanied by a decrease in strength in any one direction when compared with unidirectional fiber.

**Table 1a:** Details of lab processed indirect fiber composites

Name	Composition	Types / architecture	Processing method
Vectris Launched in 1996 by Ivoclar	Matrix- BisGMA and TEGDMA(24-39 Wt %) decandioldimethacrylate UDMA - 0.3&0.1wt%. preimpregnated E &R glass - 60Wt% for pontic and around 45-50% for the other materials.	Frame, Single, and Pontic. Single and Frame are glass-fibre woven E fibers.(Mesh) VectrisPontic unidirectional R glass-fibres (Unidirectional)	Initial polymerization -1 min with light curing unit
			final polymerization -light and heat curing unit (Targis power) for 25 minutes.
FiberKor (Jeneric/Pentron)	S-glass fibers(60%) in 100% bis-GMA matrix	FibreKor 2K strips contain 2,000 individual fibers, FibreKor4K strips contain 4,000 fibres and FibreKor 16K strips contain 16,000 fibres. (Unidirectional)	Initial polymerization -light cuirng unit (alpha lightI)for 1minute followed by light-heat curing for 15 minute in(alpha lightII) 45
EverStick net (Stick tech Ltd)	E-glass fibers impregnated with PMMA.	Mesh type glass fibers	The wetting of fibers is done with stick resin and polymerization as for fiberKor.

**Table 1b:** Details of directly processed fiber composites

Name	Composition	Fiber arcitecture	Processing method
Ribbond (Ribbond)	Polyethylene	Lenoweave (cross link stitch weave)	Chair side impregnation required
Connect (Kerr)	Gas plasma treated woven polyethylene fibers.	Braid	Preimpregnated
Splint It (jeneric/pentron)	Glass Glass polyethylene	Unidirectional Weave Weave	Preimpregnated
Everstick (Stick Tech Ltd)	Glass	Unidirectional	Preimpregnated
Fiberflex Biocomp	Kevlar	Unidirectional	Chair side impregnation required
Glaspan (glaspan)	Glass	Braid	Chair side impregnation required
DVA fibers (dental/Ventures)	Polyethylene	Unidirectional	Chair side impregnation required
Fiber-splint (polydentiainc)	Glass	Weave	Chair side impregnation required

In high stress–bearing areas, a material with high flexural strength, high elastic modulus, low deformation, and high impact and fatigue resistance is required. Fiber volume, architecture, aging, and position influences both flexural strength and modulus of resin composite. Lab studies have shown that effective reinforcement is achieved only when the fibers are placed in the side where tensile stresses act.[38,41] Applying unidirectional glass fibers which are not preimpregnated or aged at the tensile side instead of polyethylene fibers improves flexural strength. Adding polyethylene fibers on the side of compression adds strength to the material.[35] The other factors that affect the modulus of FRC are the physical and chemical properties of the composite[42] and the interfacial adhesion and matching of the modulus between the fiber and the overlying veneering composite.[43] It has been suggested by some that the interfacial bonding between the polyethylene fibers and matrix is weak.[44] It has been proved that the use of resin pre-impregnated silanized glass fibers results in the best mechanical properties.[43]

The various second-generation composites are shown inTable 2.[45–56]

**Table 2:** Details of second generation IRC

Brand name	Composition	Polymerization	Key points
Artglass Launched in 1995 By Heraeusl-Kulzer	Filler- 70wt% filler of bariumsilicate glass of 0.7*µ*. Matrix- 30wt% organic resin. Additional to conventional bifunctional molecules, Artglass contains four to six functional groups which provides the opportunity for more double-bond conversions[45].	Photo-cured in a special unit using a xenon stroboscopic light (UniXS, Heraeus/ Kulzer). The system emits 4.5 watts as usable luminous power, while the emission range is between 320 and 500 nanometers. The high intensity is emitted for only 20 milliseconds, followed by 80 milliseconds of darkness.	Can be used to fabricate inlay, onlays and crowns with/without metal substrate (ranges from nickel-chromium to gold-based metals).
		This type of light exposure increases polymerization potential. The short excitation time followed by a longer period of nonexposure allows the already cured resin molecules to partially relax, and more of the nonreactive double-bond carbon groups are made available for reaction[45].	Bonding to the metal substrate is achieved by applying an acrylonitrile copolymer (Kevloc), a flexible copolymer, to the metal surface before placing and curing the restorative material[13].
Belleglass HP introduced by Belle de St. Claire in 1996	Filler-Silanatedmicrohybrid fillers of 0.6 *µ*. Base and surface composites are available which are used on dentin and enamel respectively. Five different shades of enamel composites are available. The base composite has barium glass fillers (78.7% wt and 65% volume) Surface material has borosilicate fillers which provide enhanced optical characteristics are used (74%wt and 63% volume). Resin matrix of dentin -bis-GMA, whereas, for enamel - a combination of a hydrocarbon saturated methacrylate diurethane of TEGDMA & aliphatic dimethacrylate.	Uses two different curing units. This gives the advantage of incremental buildup and resembles the natural tooth with the hard, translucent, enamel covering the more opaque and softer dentin, able to absorb the stresses. The base composite is light cured, with a conventional light cuirng unit which stabilizes the restoration during build up and reserves unreactive surfaces for bonding. The surface composite is heat cured. The polymerization is carried by heating in an oven at 140oC at 80 psi for 20 minutes. The atmosphere is maintained oxygen free and under nitrogen gas pressure[46].	The reduction in size of the filler improves the polishability and smoothness of the material. Newer composite like “Foundation” has been modified to have a filler diameter of 30 *µ* in the base composite, which will allow for further reduction in polymerization shrinkage9.
Sinfony Introduced by 3M ESPE	Fillers - ultra-fine glass or glass-ceramic powders Pyrogenic silica is also used as a microfiller. It is a form of amorphous silicon dioxide with a primary particle diameter of < 0.05 *µ*m, produced in an oxy-hydrogen gas flame.	The proprietary system consists of two polymerising units (Visio alpha, Visio beta).the Visio alpha is equipped with a halogen lamp whereas the Visio beta is equipped with four fluorescent tubes. The polymerization wavelength ranges from 400-550nm. The polymerization mode for alpha source is 15 seconds whereas that of beta source is 40°C for 15minutes[47,48] The other non proprietary unit used is Hyper LII which is a high – intensity polymerization unit equipped with two metal halide lamps. The wavelength is in the range of 250-600nm and with an intensity of 150W for 60 seconds[49].	Used for full veneering of fixed and removable prostheses on metal frameworks, for inlays / onlays, individual crowns, glass fibre reinforced bridges and for the customization of prefabricated teeth.
	Matrix-polyfunctional methacrylate monomers.	Polymerization of this material with two different light sources improves the property[50].	Pyrogenic silica has large surface area (up to 350 m2/g) and have therefore a thickening effect. They are used to control the rheological properties of the composite. The microfiller particles can insert themselves into the gaps between the macrofillers.
Targis Launched in 1996 by Ivoclar Vivadent	[ceromer] filler- 77wt%, trimodal and has barium glass of particle size of 1*µ*. Spheroid silica filler -0.25 *µ* and colloidal silica – 0.015-0.05 *µ*. Matrix- conventional monomers.	Targis is coated with glycerin gel (Targis Gel) to prevent formation of oxygen-inhibited surface layer and placed in the curing unit Targis Power (IvoclarVivadent) for the following cycle: light emission in the first 10 min along with increase of temperature to 95°C for 25mins, and cooling for 5 min.	Targis is a veneering composite material. The material can be without framework material, to fabricate adhesive inlays/onlays/ veneers and anterior crowns. In addition, Targis is suitable for veneering metal frameworks.
SR Adoro (Ivoclar Vivadent)	The dentin and enamel materials constitute the main components.		
	components of this system include SR Link (to bond to metal frame work), a liner, dentin material, stains, incisal material and Opaquer. SR Link comprises a monomer that contains a highly hydrophobic aliphatic hydrocarbon chain and a phosphoric ester with a methacrylate function. Matrix-of dentin and incisal material consists of UDMA instead of Bis GMA and TEGDMA and the copolymer filler load is about 63% by weight. A copolymer is produced by grinding a microfilled composite into particles of approximately 10-30 *µ*m and later incorporated into inorganic microfillers. Upon polymerization, the copolymers become completely integrated into the composite and a homogeneous composite with a high loading of inorganic microfillers is obtained. The liner has 49% by weight barium glass filler particles.		Targis system has continuously been revised and the, application could now be defined for SR Adoro The phosphoric acid group of the molecule is a strong acid, which reacts with the metal or the metal oxide, forming a phosphate. The phosphates form a passivating layer on the metal surface. After the metal oxide reaction has been completed, the layer becomes very inert. The methacrylate group of the phosphoric acid reacts with the monomer components of SR Link, forming a copolymer and thereby providing a bond to the veneering resin.
Solidex Introduced by Shofu	Light cured indirect ceramic polymer system. Filler -53 vol% of 1*µ* silicon dioxide and aluminium oxide inorganic fillers[51] and ceramic microfilaments, Matrix-25 wt % co-polymers of multi-functional resins and 22% conventional resins/ light-initiators. It is available as metal primers, cervical, incisal, body, opaque and translucent shades.	The additional light polymerization is done with Solidilite system which is equipped with 4 halogen lamps for fast curing for a curing time of 1~5 minutes at a wavelength of 420-480 nm and temperature of 55 °C. Sublitecuring system is designed for initial or short polymerization during build-up without removing the restoration from the model.	
Sculpture plus (Pentron)	Nano-hybrid IRC available as body, incisal, opaceous Body and neck Paste.		
	Matrix -difunctionalmethacrylates of PCBisGMA, EBPADMA, BisGMA, UDMA and HDDMA. Fillers-silanated fillers such as barium boro-silicate glass, nano-particulated silica, zirconium silicate, photoinitiator, accelerator, stabilizer and pigments. It also contains a small amount of Al2O3.	Sculpture curing light is an automatic curing light under pressure both prior to and during light cure. The two curing cycles are a build-up cycle and a final cycle when the restoration build-up is complete. It pressurizes with nitrogen gas and automatically runs an 8min cure cycle that includes 5min of pressure, followed by 3 minutes of high intensity light.	
TESCERA ATL (BISCO INC)	Filler-The dentin material is a highly filled hybrid (85% by weight, 73% by volume). The body and the incisal material consist of a reinforced microfill (70% by weight). Added to the nanoparticles is a “reinforcement” particle that averages 1-*µ*m in size, compared to the main filler, which is 0.04*µ*m in size. The average particle size for this composite is approximately 50 nanometers (0.05*µ*m). Matrices for the dentin, body and incisal material -Bis- GMA, UDMA, ethoxylatedbis “a” dimethacrylate, and TEGDMA[52]. Incisal Material Matrix utilizes a low Bis- GMA concentration, whereas the dentin and body materials have a higher concentration	Polymerization is done in a light cup and heat cup underwater. The artificial dentin is initially pressurized (60 pounds per square inch [psi]) in a light cup before the light-curing cycle is initiated to eliminate the incorporation of internal voids and bubbles during the incremental build-up process. The light cup contains white reflection beads, which provide support to the working die while reflecting and diffusing light around the chamber and onto the composite surface. Each increment is light cured for 2 minutes. Secondary ppolymerization - heat cup with the restoration submerged in water.	This system maintains a higher density of inorganic ceramic microfillers compared to the earlier-generation direct and indirect systems[54]. These materials have the advantages of both composite resins and porcelains without being confined by the inherent limitations of either. The filler particles are silanated for suitable adhesion to the organic matrix. The presence of these 1-1*µ*m reinforcement particles - acts as a “crack arrester,” while the increased particle concentration of the microfill particles provides improved clinical performance.
		Residual free oxygen in the water is removed by adding an oxygen-scavenger agent. The final restorations are cured using an initial full cycle of pressure (60 psi) with light and heat (peak heat of 130°C and decreasing to approximately 90°C before releasing pressure) for 10 to 13 minutes[53].	
Paradigm MZ100 (3M ESPE)	85 wt% ultrafine zirconia-silica ceramic particles that reinforce a highly crosslinked polymeric matrix. The polymer matrix consists of bisGMA and TEGDMA and a ternary initiator system. The particles have a spherical shape, and an average particle size of 0.6 micrometer. This contrasts sharply with milled glass fillers in conventional hybrid composites.	Made from Z100 restorative material under optimized process conditions that assure thorough cure and a high degree of crosslinking. Paradigm MZ100 blocks are made in two cylindrical sizes, 10 and 14; these correspond to the CEREC sizes.	Alternative to porcelain blocks for CEREC restorations.
			The ultrafine zirconia-silica filler particles are synthesized by a patented sol-gel process that results in a unique structure of nanocrystalline zirconia dispersed in amorphous silica.
Vita ZetaLC (Vita Zahnfabrik)	Matrix -Bis GMA, UDMA, TEGDMA Fillers. –multiphase feldspar frits and silicon dioxide (44.3 wt%)	Additional light curing can be done with Dentacolor XS curing unit at circa 40oC at wavelength of 350-500nm	Used for the full and partial veneering of crowns, and as long-term temporary metal-free restorations. nano-sized fillers that ensure high translucency due to natural refraction.
Pearleste E2 (Tokuyama Dental Corp)	Bis-MPEPP, TEGDMA, UDMA, Filler-SilicaZirconia(0.04*µ*), Silica –titania (0.08*µ*)	Pearlcure light –high pressure mercury lamp 150W*1, 350-550nm for 120S. Pearlcure heat- heat oven 15 min under atmospheric pressure.	
Estenia C&B (Kuraray)	Matrix– UDMA, Filler –alumina ultrafine filler, glass filler (92wt%)	Secondary Light cure – Alpha II for 5 min Secondary heat cure – KL 100at 110oC for 15 minutes[55].	
Gradia (GC Corp)	Matrix- UDMA, Filler – silica powder, silicate glass powder, prepolymerised filler (75wt%)[56]	Secondary Light cure – Alpha II for 5 min	
			
			
			

**Table 3:** Comparison of properties of Second generation IRC

Name	Compressive strength (MPa)	Tensile strength (MPa)	Flexural strength (MPa)	Wear (Vol. Loss mm^3^ or Rate *µ* -)	Elastic modulus GPa	Hardness	Solubility g/mm^3^
Belleglass	163	413(d), 442(e)	142 (D) 148 (E)	0.2mm^3^ 0.35 *µ*/rev	22, 13100(d), 9700(e)	90.7	-2.5
Artglass	223kgf	-	95-130	22 mm3(after water immersion) 0.46 *µ*/rev	11	55v	-
Targis	163kgf	33(DTS)	110 -135	6 *µ*/yr 0.46 *µ*/rev	20	72	-
Sinfony*	272	-	100	26mm^3^	3	34.5	2.7
Paradigm Z100*	500	110	145	1 *µ*	8	-	-
Sculpture	281	-	140*	9*µ*/yr 0.6mm^3^	13.3*	-	3.1
Gradia	-	-	120*	0.7mm^3^	-	-	-2.1
Vita zetallc	-	-	120*	-	6	28	-
Solidex	206	37(DTS)	77	41mm(after water immersion)	16	52	-
SR Adoro	-	-	130*	0.8-1	7000MPA*	490	-

* denotes manufacturer details

## PROPERTIES OF SECOND-GENERATION IRCS

The mechanical properties of secon-generation IRCs, as assessed in various studies,[9,29,51,57–63] are presented in Table 3.

### Mechanical properties

The additional cure and the increased volume of inorganicfillers has improved flexural strength to 120 -160 MPa and elastic modulus to 8.5–12 GPa. An improvement in the degree of conversion itself does not necessarily result in better mechanical properties, because there are other factors involved, such as resin composition, filler content, and particle size and distribution. Filler content could be an important factor in deciding the physical and mechanical properties of different composite materials. Chung *et al*.[64] observed a positive relation between the volume fraction of filler and diametral tensile strength and hardness. But no correlation was observed between the degree of conversion and the mechanical properties evaluated. Neves *et al*.[65] also concluded that the filler content directly affects the hardness values. Other studies also investigated the association between the mechanical properties of composites and the filler volume. The authors reported that materials with higher filler volumes showed better mechanical properties.[66,67] Borba *et al*. observed that the hardness and flexural strength of direct resin composites were better than that of the IRCs. This was attributed to the high filler content of 78-84 wt% of D250^®^ and D350^®^ than Sinfony^®^ and Vita^®^. Thus, IRCs with lower percentage of inorganic content (e.g., Sinfony^®^, Vita Zeta^®^, with 50 wt% and 45–48 wt%, respectively) and lower values for the mechanical properties evaluated than expected for second-generation systems could be classified as *intermediate laboratory composite resins*[68]. Miranda *et al*. observed that Targis^®^ had the highest microhardness among the IRCs even though its filler content was less than in the others. This may be because there is a correlation between the method of polymerization and the microhardness. Tanoue *et al*.[69] pointed out that the best mechanical and physical properties are achieved by using a combination of composite material and curing unit from the same manufacturer. Yamaga *et al*.[70] reported that heat might facilitate monomer conversion by breaking the double bonds on the polymer network into single bonds, thus optimizing the polymerization of the residual monomers. IRCs polymerized under light activation only may have intermediate mean microhardness values (e.g., Artglass^®^ and Solidex^®^). On the other hand, Sinfony^®^ presents inferior mechanical properties, even though it is polymerized with light and vacuum. This suggests that the composition of the material influences the degree of conversion during polymerization resulting into lower resistance to indentation.[71]

Wear of composite resin materials has been evaluated in terms of two main clinical components: occlusal contact/attrition wear and contact free/abrasive wear. Filler size, volume, shape, and bonding to matrix affects wear. The chemical treatment of filler to increase bonding to matrix decreases wear.[72] Bayne *et al*. studied the wear rates and proved that the wear of Concept^®^ was less than that of belleGlass^®^. This could be due to the use of microfillers and the small particle size and the interparticle spacing, which resists wear. Belleglass^®^ showed less wear than Artglass^®^ and Targis^®^, which may be attributable to the volume of filler.[73]

Krecji and colleagues demonstrated that Artglass^®^ was considerably more wear-resistant than conventional light-cured composite resins. Charisma^®^, a conventional composite resin, exhibits an average annual wear rate of only 8 *µ*, while the Artglass^®^ formulation exhibits only 50%–60% this amount. The substantial increase in wear resistance of the indirect material can be attributed in part to the incorporation of multifunctional monomers, which permits better control over the positions along the carbon chain where the cross-linking does occur. Consequently, this can aid in improving the wear resistance and the other physical and mechanical properties of the resin matrix.[45] A change in concentration of Bis-GMA can also improve the wear resistance.

Faria *et al*. observed that the wear resistance and hardness of Artglass^®^ detroriates on immersion in water, whereas that of Solidex^®^ does not.[61] Freund and Munksgaard have found that there is a hydrolytic action of the esterase enzyme on resin restorations in the oral environment.

### Optical properties

One of the problems associated with composite materials is the unpredictable color stability. The mode of curing and the remaining double bonds may influence the color stability of the material. Nakazawa *et al*. observed that Sinfony^®^, when cured with the manufacturer-prescribed curing unit, did not discolor when immersed in water but showed color deterioration when immersed in tea. This was because of the number of remaining double bonds. On the other hand, when Sinfony^®^ was cured with the Hyper LII^®^ unit, the mechanical properties increased but it showed yellowish discoloration even on immersion in water. This is because degradation of the material may have occurred due to the heat generated by the high level of light energy.[74] Kim *et al*. also observed that there is a net color change of belleGlass^®^ during curing that should be taken into consideration when shade matching. The curing of uncured material on the tooth with a hand-held curing unit has to be done for enhanced shade matching of IRCs.[75] Papadopoulos *et al*. observed that there was an increase in lightness and a green-yellow or green-blue shift in color in IRCs on curing as well as after aging in various environments, but the changes were found to be within the clinically acceptable range.[76]

### Marginal adaptation and microleakage

Leinfelder *et al*. observed that heat-treated inlays showed less microleakage than direct restorations. Similar observations were found in other studies.[77,78] However, a few other studies found no significant differences in microleakage after thermocycling of direct and indirect resin restorations.[79,80] Aggarwal *et al*. observed that marginal adaptation and bond strength of an indirect resin system after thermocycling was better than that after direct restoration.[81] IRCs shows better marginal adaptation than ceramics because of lower polymerization contraction. The refractory die is fractured to remove the ceramic inlays and this may result in marginal microfracture, thus increasing the marginal gap.[82] Although ceramic inlays perform poorly in lab analysis, composite inlays tend to degrade in the oral environment, which can result in similar clinical behavior of both the materials

### Surface properties

One of the main failures of IRC restoration is the formation of secondary caries due to plaque accumulation, which is aggravated by the surface roughness of the material. The biofilm accumulation is based on the filler size and matrix monomer. Smaller filler size with more weight% produces a smooth surface and, consequently, less biofilm adhesion. The surface roughness ranges from 6–8 *µ*. Polishing with diamond pastes also renders a smooth surface. Another possible factor for bacterial adherence is the presence of remaining uncured monomers.[56]

### Surface treatment of IRCs

The treatment of the intaglio surface of indirect restorations determines the bonding of the restoration to the tooth. The use of hydrofluoric acid for surface treatment causes microstructural alteration of the composite because of the dissolution of the inorganic particles.[83] The best alternative method to raise the surface energy is by sand-blasting with aluminium oxide particles for 10 sec.[20] This causes a non-selective degradation of the resin and promotes better adhesion. According to Soares, application of silane after sand-blasting resulted in higher bond strength. Since the compositions of the IRCs are similar, the surface treatment for all materials can be the same.[84] The various clinical studies comparing the materials are tabulated in Table 4.[85–98]

**Table 4:** Summary of clinical studies on IRC.

Clinical study type	Materials compared	Parameters compared	Comments
Four – six year follow up of resin inlay /onlay[85] (Leirskar *et al*. 2003)	Tetric, Maxxim, Z 100	Form, MF, MD, CM, SR, SC	22% of restorations - acceptable and required minimal corrections.
			2- surface restoration performed better than 3 surfaced and onlays
Seven year follow up resin inlay / onlay[86] (Donly *et al*. 1999)	Cast gold, Concept	F, CM, MD, SC, W, SR, (Ryge criteria) in molars and premolars	50-75% Alpha ratings for all parameters for concept. Concept yields clinically acceptable restorations particularly in premolars
One year follow up[87] (Cetin AR *et al*. 2009)	AELITE, Filtek supreme XT, Tetricevoceram (Nano filled direct composites) Tescera ATL, estenia	Class I and Class II SR, CM, MD, PS, GA, Rt (Ryge criteria)	85-100% Alpha ratings for all parameters for both direct and indirect composites.
Three year follow up[88] (Ducik w *et al*. 2010)	Admira (ormocer), Grandio	SR, F, MI, MD (Modified USPHS criteria)	Alpha 1 score for overall success -71% - both materials Both materials have acceptable success after 36 months
Three yr follow up of CAD CAM composite and ceramic[89] (Vanoorbeek *et al*. 2010)		marginal fit, periodontal parameters, volume loss, and wear patterns of the veneering material	Survival rate andsuccess rate was 88 and 56% forcomposite
			Survival rate andsuccess rate was 97 and 81% for ceramics
			Increased wear and decreased esthetics of composite makes ceramics superior for CAD CAM restorations
One year results for direct and indirect composite inlays[90] (Mendonca *et al*. 2010)	TetricEvo Ceram, Targis	modified USPHS criteria forCM, MD, SC, AF, SR, MI, PS.	This is a short term study.
			CS, PS – no changes
			CM, AF, SR similar to both materials.
			Other parameters direct resin performed better.
A 4-6 yr retrospective study on cracked tooth bonded with indirect resin[91] (Signore *et al*. 2007)	Direct composite restoration followed by indirect onlay		Acceptable &survival rate-93 % 7% failure rate
A 3 yr RCT in evaluating direct and indirect composite for severely worn teeth[92] (Bartlett 2007)	32 direct and indirect restorations in premolar and molar of severely worn teeth.	Wear fracture and loss of material was evaluated	22% fractured, 28% - complete loss of restoration. Direct and indirect resin composites for restoring worn posterior teeth is contraindicated.
10 year follow up on direct, indirect and ceramic inlay[93] (Thordruo 2006)	Cerec Cos 2, Vita Dur, Brilliant DI, Estilux	modified retrospective study on cracked tooth bonded with indirect resin (Signore et.al 2007California Dental Association Quality Evaluation System SR, F, SC	3 estilux inlays were replaced
			6 vitadur inlays were repaired
			Both materials were in acceptable range after 10 yrs.
5 yr follow up of direct inlays and conventional resin restorations[94] (Wassell *et al*. 2002)		MD, MF, SR, W (USPHS criteria)	More failure of inlays than conventional composites Direct inlay technique gave no clinical advantage over conventional, incremental placement
Composite resin fillings and inlays. An 11-year evaluation[95] (Pallesen *et al*. 2003).	Brillinat DI, Estilux, SR isosit	Class II restoration in molar and premolars (USPHS criteria)	70% of direct fillings and 88% of inlays were in acceptable ratings
			Failure more in molar than premolar failure were fracture of restoration, secondary caries, fracture of tooth, loss of proximal contact, and loss of restoration
			no significant difference between fillings and inlays or between types of restoration
Three year follow up of resin inlays[96] Manhart *et al*. 2010)	Artglass, Charisma	Class I, single and multisurface Class II inlays. (USPHS criteria)	89.8% of Artglass and 84.1% of Charisma inlays – acceptable
			No significant differences between premolar and molars
			Small inlays survived better
			Failure was mainly due to bulk fracture, loss of marginal integrity.
Two year wear assessment[97] (Stober *et al*. 2008)	Artglass	Influence of gender, arch, and crown location on the occlusal wear	In 1 yr – 19*µ* for anterior and premolar, 21*µ* for molars
			In 2 yr – 36 *µ* for anterior, 44*µ* for premolar and 84*µ*
			In 2 yr – 36 *µ* for anterior, 44*µ* for premolar and 84*µ* for molars.
Adhesively luted metal free crowns for5 years[98] (Lehmann *et al*. 2009) Artglass	Artglass	Location and preparation design on survival rates. 68 posterior, 46 anterior crowns with 0.5mm shoulder / 0.5mm chamfer was prepared	13 crowns – replaced, 5 crowns – repaired. No difference between location or preparation design on complications.
			survival rate -96% in 3 yrs 88.5% in 5 yrs can be used as long term temporary crown increased plaque accumulation restricts the indication

MD- marginal discoloration, SC – secondary caries, SR- surface roughness, MF- marginal fit, W-wear, CM- color match, F- form, MI-marginal integrity.

### Clinical advantages of IRCs

A properly fabricated indirect restoration is wear resistant, esthetic, and relatively less prone to postoperative sensitivity. Since, the only polymerization that occurs is that associated with a thin liner of luting agent, the potential for tensile stresses on the odontoblastic processes is considerably less, which translates into less potential for postoperative sensitivity. Indirect laboratory-processed composite resin systems provide an esthetic alternative for intracoronal posterior restorations and may also reinforce tooth structure. IRC restorations offer some benefits as compared to direct restorations, such as better mechanical performance and a significant reduction in polymerization shrinkage (i.e., limited to the dual-cured luting cement).[2,22] Additional clinical benefits include precise marginal integrity, ideal proximal contacts, excellent anatomic morphology, and optimal esthetics.[21]

When compared to porcelain and porcelain-fused-to-metal restorations, the transfer of masticatory forces is considerably less. Composite materials have shown a greater capacity to absorb compressive loading forces and reduce the impact forces by 57% more than porcelain. Thus, a polymer of the above-mentioned materials is considered when restoring the coronal aspect of a dental implant. It has been shown that the edge strength of belleGlass^®^, either alone or with fiber reinforcement, is more than that of ceramics. This reflects the ability of the material to maintain the marginal integrity to occlusal loading.[99] Tsitrou found that resin composites have a lower tendency for marginal chipping than ceramics.[100] Due to the similar composition of the luting cement and composites, the marginal adaptation of composites is better than that of ceramics

## CONCLUSION

Our literature review shows that there are numerous IRCs available nowadays. These materials perform well in *In vitro* and short-term *In vivo* studies. It is also apparent that IRCs can effectively supplement the use of ceramics in certain clinical conditions. The improvement in properties due to the additional polymerization, which was observed in these studies, needs to be assessed with long-term clinical trials. In the absence of multiple long-term studies, the survival rate of IRC restorations cannot be assessed. Further clinical research is needed to evaluate the success rates with these newer IRCs.
